# Stepwise disassembly of supramolecular structures triggered by specific protein binding

**DOI:** 10.1016/j.bpj.2025.11.012

**Published:** 2025-11-12

**Authors:** Zhiguang Jia, Allen M. Chen, Shanlong Li, S. Thayumanavan

**Affiliations:** 1 Department of Chemistry, University of Massachusetts, Amherst, Massachusetts; 2 Northfield Mount Hermon, One Lamplighter Way, Mount Hermon, Gill, Massachusetts; 3 Department of Biomedical Engineering, University of Massachusetts, Amherst, Massachusetts

## Abstract

Supramolecular assemblies that can spontaneously disassemble in response to specific protein binding provide a powerful platform for sensing and targeted delivery. This concept has been previously demonstrated using a peptide-based amphiphilic polymer (P1) that consists of both hydrophobic M1 and hydrophilic M2 and M3 side chains and presents a specific ligand for protein bovine carbonic anhydrase II (bCA-II). To further understand the molecular forces and mechanism of the assembly and disassembly of P1, we developed a coarse-grained modeling framework for direct simulation of the dynamic polymer-unimer equilibrium of amphiphilic peptide nanoassembly. The results show that P1 unimers initially self-assemble into small micelles, which are capable of encapsulating hydrophobic cargos such as the dye molecule DiI. The micelles subsequently aggregate into larger multicore nanostructures. Notably, the micelle architecture persists within the larger aggregates, with the hydrophobic M1 side chains forming distinct cores. The simulations further show that introduction and binding of bCA-II to the M3 side chains, which contain the specific ligand, lead to a disassembly process. Our simulations revealed that bCA-II binding causes a stepwise disassembly of the aggregates. First, the micelle-micelle interfaces are replaced by micelle-water interfaces, with minimal exposure of individual M1-concentrated hydrophobic cores. Complete disassembly occurs only when multiple bCA-II molecules bind to a single micelle, leading to the breakdown of the hydrophobic core and the subsequent release of encapsulated DiI molecules. This stepwise disassembly mechanism underscores the intricate balance between structural stability and responsiveness in these supramolecular assemblies. Our findings provide new insights into the design of smart materials for biomedical applications, where controlled release mechanisms are essential. The ability to fine-tune the assembly and disassembly processes through specific molecular interactions opens many new possibilities for the development of responsive drug delivery systems and biosensors.

## Significance

The ability to precisely recognize and respond to noncovalent binding of specific proteins in supramolecular nanoassemblies has important applications in biosensing, targeted delivery, and more. This work describes a coarse-grained molecular modeling framework for direct simulation of the self-assembly of amphiphilic polypeptide-based polymers as well as their disassembly process in response to binding of a specific protein. The results provide key insights into the molecular organization, driving forces, and mechanism of self-assembly and disassembly of these polymers, paving the way for rational design of new polymers with improved sensitivity and specificity as well as robustness in operation in complex environments.

## Introduction

Developing “smart” materials for various biomedical applications, such as targeted drug delivery, biosensing, and tissue engineering, has long been a major interest in supramolecular chemistry. Specifically, nanoassemblies that encapsulate guest molecules under one set of conditions and then disassemble and release the guest molecules under different specific conditions are considered highly promising due to their high-performance targeting and tunable local release capabilities (1,2,3,4,5,6,7). Triggers that can induce spontaneous disassembly include recognition of signal molecules or micro-environmental change such as pH, light, and temperature (8,9,10,11,12,13,14). Fundamental investigations into the design principles of self-assembly and disassembly, especially through mechanisms with more specificity than traditional triggers, could greatly benefit both biomedical research and clinical applications on this front.

**Figure 1:**
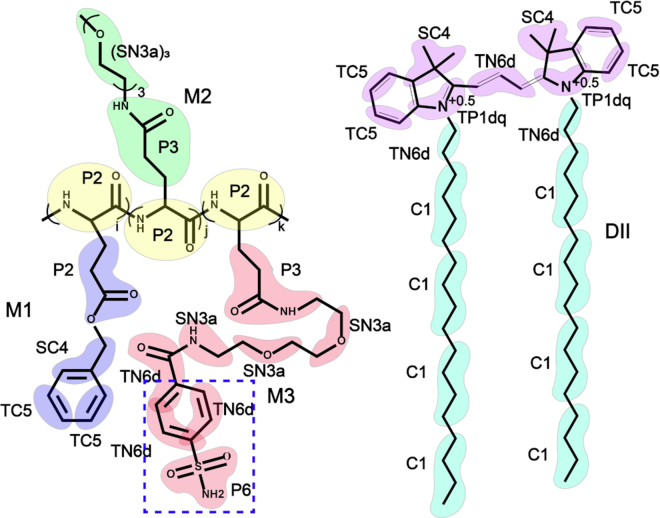
Chemical structures and all-atom-to-coarse-grained (AA-CG) mapping of P1 polymers with M1, M2, and M3 monomers (*left*) and the dye molecule DiI (*right*). Clusters of circled atoms are mapped to the same CG beads and labeled with the corresponding Martini bead types. Overlapping circles indicate atoms contributing to multiple beads. The benzene-sulfonamide ligand in M3 for specific bCA-II binding is highlighted with a blue rectangle. The positive charge in the DiI carbocyanine group is divided into two 0.5 partial charges to reflect the resonant charge distribution. Note that P1 polymers are random copolymers.

In previous work, we described a system of amphiphilic polypeptides, referred to as P1 polypeptides, which forms nanoscale assemblies that are stable under normal physiological conditions but disassemble in the presence of specific proteins that recognize ligands covalently attached to the side chains (15). This system’s ability to encapsulate and release guest molecules under specific conditions, its noncytotoxic properties, and the simplicity of its synthetic route make it highly promising for applications in drug delivery, diagnostics, and biosensing. Specifically, the molecular design of this system is based on three different substituted glutamic acid monomers, denoted as M1, M2, and M3 (Fig. 1). The side chain of M1 is hydrophobic, whereas the side chains of M2 and M3 are hydrophilic. Oligoethylene glycol (OEG) moieties are added to M2 and M3 to minimize nonspecific protein interactions. Additionally, a benzene-sulfonamide group is attached to the M3 side chain, enabling specific binding to bovine carbonic anhydrase II (bCA-II).

Transmission electron microscopy and dynamic light scattering analyses suggest that the mature P1 assemblies adopt a spherical structure with a hydrodynamic diameter of approximately 170–200 nm (15). However, the molecular structure and organization of these assemblies remain unclear. The small hydrophobic side chain of M1 makes it less likely to form a single micelle; instead, it might organize into a large liposome-like structures or complex multicore assemblies. Additionally, the mechanism by which protein binding to M3 triggers the disassembly of the supermolecule is not fully understood. Potential mechanisms could involve the solvent exposure of the hydrophobic core, surface remodeling, competition between P1-P1 and P1-protein interactions, and/or structural frustration within the assembly. Better understanding of the structural dynamics of these nanoassemblies could be instrumental to rational design and advancing applications in drug delivery, biomarkers, or responsive nanomaterials.

In this work, we aim to develop a coarse-grained (CG) molecular modeling and simulation framework for elucidating the driving force and molecular mechanism of self-assembly and noncovalent protein binding-induced disassembly of P1 amphiphilic polypeptides. Specifically, the simulations suggest that unimers initially aggregate into small micelles, capable of encapsulating hydrophobic cargos, which then further assemble into larger multicore supramolecular nanoparticles. Notably, the small micelle architecture is largely preserved within the mature nanoassemblies, despite partial mixing with neighboring micelles and the connection of their hydrophobic cores. Furthermore, we show that specific protein binding induces a stepwise disassembly of the nanoparticle, beginning with the breakdown of interactions between micelles, leading to the formation of smaller aggregates. Further increasing the protein concentration promotes the disassembly of these smaller aggregates into protein-bound unimers and multimers, during which the cargo is released. These findings lay the groundwork for future experimental and computational studies aimed at rational engineering of the polymer design, such as to achieve desired release kinetics or to develop multiple signal-triggered sensing and disassembly.

## Materials and methods

### Coarse-grained modeling of P1 polymers, cargo, and proteins

The CG model for P1 polymers was constructed using the Martini 3 CG force field (16,17). The Martini framework is uniquely suitable for these polypeptides because it has been carefully balanced based on thermodynamic data on partition and provides a general scheme for introducing new chemical motifs. The mapping from all-atom (AA) to CG topology and the corresponding Martini bead types were assigned by identifying similar small molecules within the Martini 3 force field. Regular beads, small beads, and tiny beads were mapped four-to-one (four atoms per one bead), three-to-one, and two-to-one, respectively. The martinize2 was used for initial conversion of energy-minimized AA structure to CG model (18). The backbone is disordered in the polymer, and all backbone beads were thus assigned the coil-type beads (P2). The sulfonamide group in M3 has no relevant analog present in current Martini 3 force field; it was represented using a single highly polar P6 bead, which has been used to represent sulfoxide in Martini 3 (17). This bead was chosen due to it having a similar octanol to water transfer free energy (∼−12 kcal/mol) as its highly water soluble analog sulfamide (17). The final models of P1 polymers as well as the dye molecule Dil are summarized in Fig. 1.

Missing bonded parameters were derived by first calculating the corresponding distances from energy-minimized AA structures and then selecting the closest bond in chemical similar groups, with angle and dihedral parameters being taken from same group. Although the majority of bead types adhere to standard Martini 3 guidelines, several exceptions were introduced to better reflect the unique chemical properties (such as resonance structures and highly hydrophilic substituent groups) as well as design rationale specific to the P1 polymer system. Specifically, for the M3 monomer, the secondary amide linked to the backbone was assigned a P3 bead type. Even though the Martini 3 guidelines generally recommend a P2 bead for secondary amides, this assignment makes it more hydrophilic and thus indistinguishable from the ester linker in the M1 monomer, which is assigned the P2 bead type per Martini 3 standard. Additionally, we employed a six-to-one heavy-atom-to-bead mapping for this particular bead to preserve the integrity of the subsequent polyethylene glycol segment, designed specifically to form a stable hydrophilic layer. Another nonstandard CG mapping is where the secondary amide group attached to the benzene-sulfonamide moiety is split into two separate beads. This allowed the carbonyl group to be associated directly with the benzene-sulfonamide to better reflect the actual conjugation. For the benzene-sulfonamide group itself, we utilized a TN6d bead to reflect its increased hydrophilicity, mimicking the most polar bead within the histidine side-chain imidazole ring. A similar rationale underlies the bead choices for the DiI cargo molecule: we assigned a more polar bead type (TN6d) to the conjugated linker region and the first bead of the alkyl tail, diverging from standard Martini choices. These choices reflect the higher polarity and resonance stabilization of these specific groups, aiming to better capture the molecular characteristics relevant to the system’s behavior. In addition, to examine the robustness of our model, we developed an alternative set of force field parameters that strictly follow the standard Martini 3 CG bead typing guidelines (Fig. S1). Using this alternative parameterization, we conducted additional simulations of the self-assembly, cargo embedding, and specific protein-binding-induced disassembly processes. The results were then directly compared with those obtained from the original model containing a small number of nonstandard bead assignments (Figs. S5 and S6).

The initial AA structures were generated using the program CHARMM (19). Four polymers with randomly generated sequences of M1, M2, and M3 were constructed with optimal internal coordinates. Each P1 polymer contains five to six M1, four M2, and one to two M3 monomers, which is consistent with the molecular weight (3.2 ± 1.2 kDa or 11 ± 4 monomers) and molar ratio of 0.48:0.37:0.15 reported in the previous experimental study (15). The sequence of all four peptides is listed in Table S1. These atomistic structures were then transformed into CG conformations using the Martinize tool (20). The initial structures of bCA-II, bovine serum albumin (BSA), and lysozyme were taken from PDB: 1v9e, 4f5s, and 7lzm, respectively (21,22,23). For bCA-II, the Zn coordination site involving histidine residues (H93, H95, and H188) was modeled by introducing additional bonded parameters with distances set to 2.4 Å. Currently, the Martini force field does not provide a default bead type for Zn; thus, a bead type typically used for Ca^2+^ ions (type SD) was employed to represent the Zn ions. To reproduce the specific interactions between bCA-II and the benzene-sulfonamide ligand in M3, the σ and ε in Lennard-Jones terms between the ZN ion bead in bCA-II and terminal P6 beads in M3 (Fig. 1) were set to 5 Å and 15 kcal/mol, which are comparable to the distance observed in the bound structure (4–6 Å) and binding free energy (10–12 kcal/mol for benzene sulfonamides analogs) (24,25). The online version contains supplementary material available at https://github.com/zhiguangjia/MURI_self_disassemble_P1.

### Molecular simulation of self-assembly and disassembly

All simulations were performed using CUDA-enabled versions of Gromacs 2020 (26,27). The self-assembly, cargo encapsulation, and binding-induced disassembly were studied. Simulations for each of these three phases were run separately, where latter simulations utilized the final structures of the aggregates from previous simulations. For self-assembly simulations, the randomly sequenced P1 polymers (ratio 2:1:1:1) (Table S1) were initialized at random locations within the simulation box (Table S2, *sim 1–6*). For subsequent simulations of loading DiI cargo, the final P1 assemblies from simulations 1–6 were placed in the center of the box, and DiI was inserted at random positions within the simulation box (Table S1, *sim 7–8*). For the simulations of bCA-II binding-induced disassembly, bCA-II proteins were placed at random positions near the assemblies in such a way that the orientation and position of bCA-II kept its Zn atom within 5–10 Å of the benzene-sulfonamide bead in each M3 ligand (Table S1, *sim 9–12)*. To minimize self-aggregation of proteins, which is a known drawback of the Martini model, the strength of interactions between the protein (BSA, lysozyme, and bCA-II) and water was increased by 10%, as reported previously (28). Specifically, all protein beads (e.g., TP1) were duplicated and renamed (e.g., WTP1). The corresponding nonbonded Lennard-Jones parameters in the [nonbond_params] section of the Martini parameter files were duplicated accordingly (e.g., TP1-TP1 duplicated as WTP1-WTP1 and WTP1-TP1). Subsequently, the Lennard-Jones ε parameter between these newly defined protein beads and the water (W) beads was increased by 10%.

All systems were solvated with Martini CG water and neutralized with counter ions. The solvated systems were first minimized for 5000 steps using the steepest descent algorithm, followed by 200 ps of equilibration steps where the positions of heavy atoms were harmonically restrained using a restrained force constant 0.1 kcal.mol^−1^ Å^−2^. Integration was performed using the leapfrog algorithm with a time step of 10 fs (29). The Verlet neighbor search algorithm was used to update the neighbor list every 20 steps (30). For the Lennard-Jones terms, a cutoff scheme with a value of 1.1 nm and the Verlet cutoff scheme (31) for the potential-shift were used. Long-range electrostatic interactions were treated with a reaction field with relative permittivity set to ε_r_ = 15 and a cutoff value of 1.1 nm (32). Temperature was maintained at 303 K using a Berendsen thermostat using time constant τ_T_ = 0.5 ps (33). The Parrinello-Rahman barostat (34) was used for pressure coupling with a reference pressure of 1 bar, 4.5 × 10^−5^ bar^−1^ compressibility, and a time constant τ_p_ = 4.0 ps. The lengths of all production simulations are summarized in Table S2.

### Simulation analysis

DBSCAN clustering analysis was performed using MDAnalysis and Sci-Learn together using in-house scripts (35). The maximum distance (eps) between two samples (peptide or M1 side chain) was set to 8 Å and the minimum number of samples (min_samples) in a neighborhood for a point to be considered as a core point was set to 5. The atomic density profiles across the aggregates were calculated as follows. First, a cylindrical region centered at the center of mass of the aggregates was defined. The diameter of the cylinder was set to 6 Å, and it was aligned along the X-axis. All atoms within this cylinder were then selected, and the density along the axis was computed using a bin size of 0.5 Å. All density profiles are derived from last 50 ns of simulations. The shape of P1 assemblies throughout the simulation trajectory was investigated by calculating two shape parameters using MDAnalysis and in-house scripts: asphericity (a) and prolateness (p) (36,37,38). These two parameters were calculated from the eigenvalues λ1, λ2, and λ3 of the radius of gyration tensor (Eq. 1),

(1) $$S_{\alpha \beta }=\sum_{i=1}^{N}\left(r_{a}^{i}-r_{a}^{com}\right)\left(r_{\beta }^{i}-r_{\beta }^{com}\right)\text{,}$$

where N is the total number of CG beads in one P1 polymer, and $r_{a}^{i}$ and $r_{a}^{com}$ are the *a*^*th*^ Cartesian coordinate of the position vector of *i*^th^ particle and the center of mass, respectively.

With the eigenvalues of the tensor λ1 < λ2 < λ3, the asphericity (a) and the prolateness (p) parameters are calculated by the following formulas:

(2) $$a=\frac{{\left(\lambda_{2}-\lambda_{1}\right)}^{2}+{\left(\lambda_{3}-\lambda_{1}\right)}^{2}+{\left(\lambda_{3}-\lambda_{2}\right)}^{2}}{{2\left({\lambda_{3}+\lambda }_{2}+\lambda_{1}\right)}^{2}}$$

(3) $$p=\frac{\left({2\lambda }_{1}-\lambda_{2}-\lambda_{3}\right)\left({2\lambda }_{2}-\lambda_{1}-\lambda_{3}\right)\left({2\lambda }_{3}-\lambda_{1}-\lambda_{2}\right)}{{2\left({{\lambda_{1}^{2}+\lambda_{2}^{2}+\lambda_{3}^{2}-\lambda }_{3}\lambda_{1}-\lambda }_{2}\lambda_{1}-\lambda_{2}\lambda_{3}\right)}^{\frac{3}{2}}}$$

As the shape of the aggregate deviates further from a perfect sphere, the asphericity approaches 1. The value of prolateness ranges between −1 and 1. For *p* = −1 the object is fully oblate (λ1 < λ2 = λ3), whereas *p* = 1 implies a perfectly prolate shape (λ1 = λ2 < λ3). All molecular illustrations were prepared using VMD (39).

## Results

### Direct simulation of the self-assembly of P1 polymers

**Figure 2:**
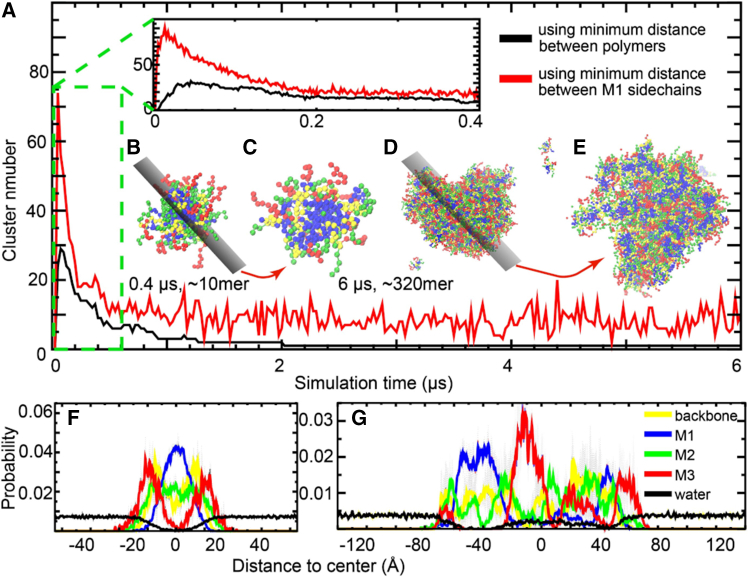
Self-assembly of P1 polymers. (*A*) Change in cluster number as a function of simulation time. The DBSCAN clustering results, using the minimum distance between peptides and M1 side chains (see materials and methods) are plotted as black and red traces, respectively. (*B*) A representative snapshot of a 10-mer micelle, with backbone colored in yellow and the side chains of M1, M2, and M3 backbones colored in blue, green, and red, respectively. (*C*) Longitudinal section through the center of aggregate in (*B*) (*indicated by the black plane*). (*D*) A representative snapshot of a 320-mer aggregates, with the same rendering style as in (*B*). (*E*) Longitudinal section through the center of aggregate shown in (*D*) (*indicated by the black plane*). (*F*) Particle distributions along a cylinder (6 Å diameter) across the center of mass of the 10-mer aggregate shown in (*B*). (*G*) Particle distributions along a cylinder (6 Å diameter) across the center of mass of the 320-mer aggregate shown in (*D*).

To investigate the self-assembling process, 10 to 320 P1 unimers were placed at random positions within the simulation box (Table S1, *sim 1–6*). In all simulated systems, these P1 unimers aggregated into a single large cluster. As shown in Fig 2 and Video S1, in the largest system, which contained 320 P1 unimers and started from a unimer state (no cluster or cluster number = 0, black trace), the unimers rapidly formed local oligomers, and the cluster number peaked at ∼80. These oligomers then aggregated into small micelles, each containing 10–20 P1 unimers, and the cluster number reduced to ∼20. The atom densities along a cross-sectional slab across the cluster center (Fig. 2, *E* and *F*) suggest the micelles adopt a three-layer structure: the M1 side chains were buried in the middle, forming a hydrophobic core (blue trace). This core was covered by the backbones of M1, M2, and M3 (yellow trace), which formed a surrounding hydrophilic shell. The side chains of M3 (red trace) were exposed on the surface, forming the outer shell. M2 side chains (green), were mixed throughout both the inner core and in the hydrophilic shell but had a lower probability than M3 of appearing in the outer shell. In these simulations, it is important to note that since M3 constitutes only 15% of the monomer units of P1 polymers, the outer shell has a relatively loose structure, with M3 side chains covering only 40% of the solvent-accessible surface area (SASA). As a result, the middle layer remains highly accessible to the solvent.

Video S1. A representative trajectory of the self-assembly of P1 polymers The movie was based on simulation 6 from Table S2. P1 polymer is colored gray, and M3 side chains are highlighted in red.

**Figure 3:**
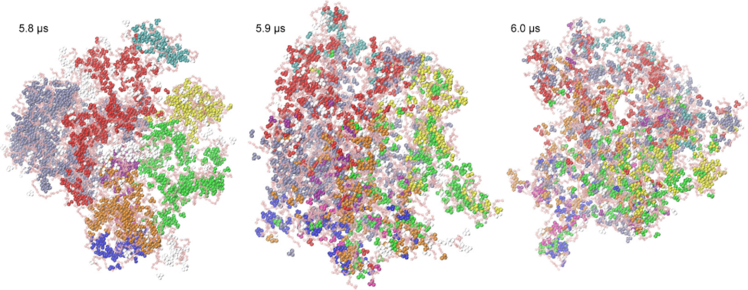
Diffusion of P1 unimers within the aggregates over time. The M1 side chains are shown as spheres, and the backbone is represented by transparent lines, with the rest of the P1 polymer omitted for clarity. The colors indicate the cluster IDs at 5.8 μs (only clusters containing more than five polypeptides are shown in colors; smaller clusters are shown in white for clarity). These snapshots were taken from *sim 6* of Table S2.

In all simulated systems, the small aggregates then merged into a single larger cluster in the later stages (cluster number = 1) (Figs. 2 and S2; Video S1). Despite variations in cluster size due to the different initial numbers of unimers in the systems, all large clusters adopted a hierarchical multicore structure. As shown in Fig. 2, the micelle structure, especially the hydrophobic core, remained distinguishable even after micelle aggregation. This feature is reflected by the side-chain distributions across the middle of a large cluster, where the presence of M1 clusters (blue traces) is reflected by multiple peaks in the profile (Fig. 2 *G*). In addition, as shown in the profile, the side chains of M3 (red traces) are not only exposed on the outside of the aggregate but are also buried inside the aggregate, bridging interactions between neighboring micelles. The side chains of M2, as observed in the single micelles, are mixed with both the hydrophobic core and hydrophilic shell of micelles. It should also be noted that, although the side-chain distribution is symmetric in small clusters (Fig. 2 *F*), this symmetry is lost upon the formation of higher-order aggregates (Fig. 2 *G*). This loss of symmetry reflects the disordered packing of small clusters within the larger aggregates. Further DBSCAN clustering analysis revealed that most individual micelles within the larger aggregates contained ∼50 to ∼200 M1 side chains (corresponding to 10–40 P1 polymers) (Fig. S3). However, a small population of large clusters containing up to 750 M1 side chains (corresponding to ∼150 P1 unimers) has also been observed (Fig. S3), which formed large hydrophobic cores across the aggregates (e.g., Fig. 3, red and cyan clusters). The hydrophilic side chains and the middle backbone layers partially merged between neighboring micelles, which may have also contributed to stability of the large aggregates. The hydrophobic cores of adjacent micelles were also connected by M1 side chains at the interfaces, and such connections produced clusters that span the full length of a single aggregate (Fig. 3 left panel, red and cyan clusters). Generally, the structure of the assemblies was highly dynamic throughout the simulation timespans, with each individual polypeptide migrating between micelles and forming variations of these aforementioned structures. For example, Fig 3 illustrates that the M1 side chains within same cluster rapidly diffuse within the aggregate and could become well mixed and reorganized to form new clusters within 0.2 μs. Therefore, nanoassemblies of these polypeptides remain not only hierarchical but also highly dynamic, which may be important properties that allow them to be responsive to binding of specific proteins.

### Encapsulation and release of the dye molecule cargo DiI

**Figure 4:**
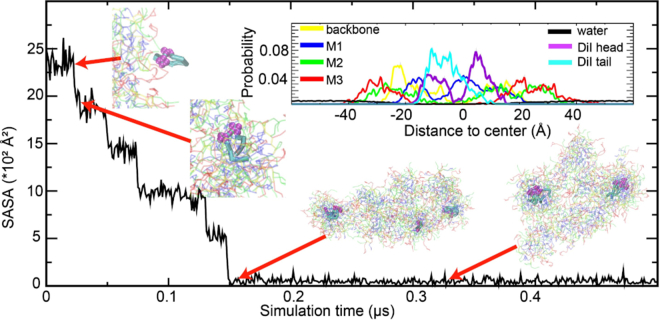
Encapsulation of DiI into P1 polymer aggregates. The solvent-accessible surface area (SASA) of the DiI headgroup is plotted as a function of time. Snapshots at 20 ns and 40 ns show the embedding of a single DiI molecule into the aggregate. Snapshots at 0.15 μs and 0.3 μs illustrate the diffusion of the DiI molecule within the aggregate. The backbone is colored yellow, and the side chains of M1, M2, and M3 backbones are colored in blue, green, and red, respectively. The head and tail of DiI are depicted as purple spheres and cyan sticks, respectively. The inset shows the particle distribution along a cylinder crossing the center of mass of one DiI aggregate at 0.5 μs.

To examine the cargo loading of P1 polymer aggregates, five DiI molecules were placed at random positions within a simulation box that contains a preformed aggregate of 80 P1 polymers (Table S1, *sim 7*). In all three simulations, all DiI molecules readily absorbed into the M1-concentrated interior of the aggregates within 500 ns, leading to the gradual decrease in the SASA of the DiI headgroups (e.g., see Fig. 4). Upon embedding, the SASA of each DiI headgroup dropped from ∼500 A^2^ to less than ∼1 A^2^, indicating that the DiI was completely isolated from water (Fig. 4). This observation is consistent with previous experimental results, which showed an increase in DiI emission after being loaded into the hydrophobic interior of P1 aggregates (15).

Because the DiI tail is more hydrophobic than the M1 side chain, the aliphatic tail forms a distinct hydrophobic core (Fig. 4 inset, cyan trace) where M1 (blue traces) is partially excluded. Interestingly, due to the small size of the micelles, the DiI headgroup (purple trace) not only appears in the M1/M2 region but also mixes with the DiI tails. The disorder observed in DiI orientation likely originates from the size mismatch between the short M1 side chains and long DiI tails, which contrasts with the behavior of DiI in lipid bilayers, where the DiI tail has a similar length to the surrounding lipid tails (40). The DiI molecules are also dynamic inside the aggregates and can transfer between the constituent micelles (Fig. 4). It should be noted that similar fast cargo exchange has been observed in previous experiments (41), which was considered an indicator for evaluating the encapsulation stability.

### Specific protein binding-induced disassembly of P1 nanoparticles

**Figure 5:**
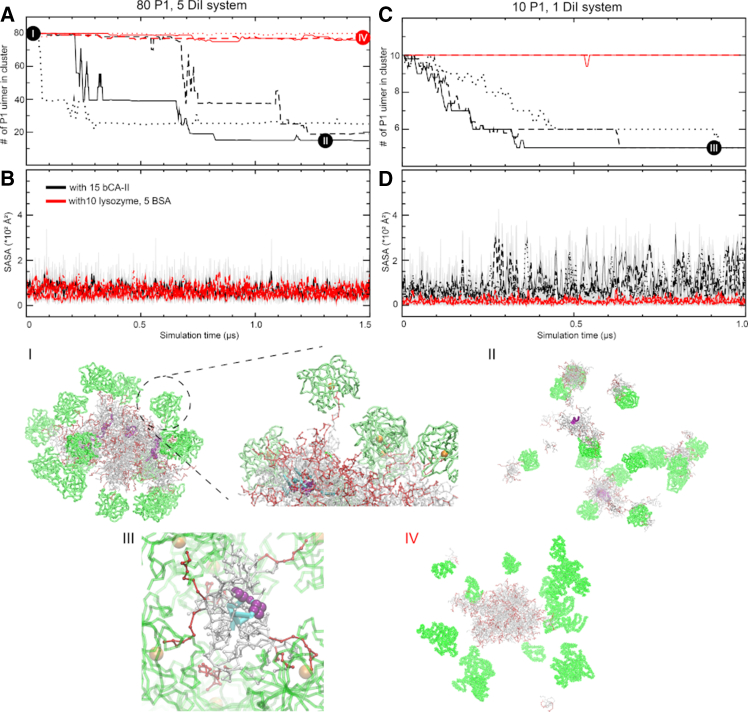
Stepwise dissemble of PI aggregates upon bCA-II binding. (*A*) Average number of P1 polymers in the aggregate and (*B*) SASA of Dil headgroup over time for a system of 80 P1 and 5 DiI. (*C*) Number of P1 polymers in a micelle and (*D*) SASA of Dil headgroup over time for a system of 10 P1 and 1 DiI. Black traces are derived from three independent simulations with 15 bCA-II (Table S2, sim 9 and 11), whereas red traces are derived from independent runs with 10 lysozyme and 5 BSA (Table S2, sim 10 and 12). The bottom row shows representative molecular structure at selected time points: (I) 0 μs and (II) 1.3 μs of 80 P1/5 DiI/15 bCA-II system, (III) 0.8 μs of 10 P1/1 DiI/15 bCA-II system, (IV) 1.5 μs of 80 P1/5 DiI/5 BSA/10 lysozyme system. A zoom-in is also shown for snapshot (I) in the circled region, showing details of binding between M3 and bCA-II. Note the exposure of DiI head to the bulk solvent in snapshot (III), and the stability of the P1 aggregates in snapshot (IV). In all snapshots, P1 polymer is colored gray, and M3 side chains are highlighted in red. External proteins are shown in green, with Zn ions on bCA-II represented as cyan spheres. DiI molecules are colored with a purple head and cyan tails.

Previous experimental studies have shown that upon the addition of bCA-II (P1:bCA mole ration 5:3), which specifically binds to the benzene-sulfonamide ligand on M3, the overall aggregate size decreases to approximately the size of the bCA-II protein itself (∼5 nm) (15). Concurrently, the DiI fluorescence became quenched by water, suggesting that the loaded cargo is either released or becomes exposed to the aqueous environment. To examine the mechanisms of the disassembly process, 15 bCA-II molecules were placed near the exposed M3 side chains of the 80-mer P1 aggregates (Table S2 *sim 9*; see materials and methods). As a negative control, a separate set of simulations was run with 10 lysozyme and five BSA protein molecules placed in the bulk solvent (sim 10). It should be noted that these two proteins were also used as the negative control in the experimental study (15). As shown in Fig. 5 *A* (red traces), in the presence of lysozyme and BSA, the number of P1 in the aggregates has little change during the simulations, which suggests that the P1 aggregates remained stable against nonspecific protein binding. In contrast, specific binding of bCA-II significantly destabilizes P1 aggregates, and the average number of P1 unimers in aggregates dropped from 80 (a single aggregate) to ∼10–20 (five to six small micelles) (Fig. 5 *A*, black trace and snapshots I and II). Note that the disassembly processes display a clear stepwise nature, with each micelle separated frequently as a unit and the aggregate size dropped discontinuously (also see Video S2). As shown in the snapshot, P1 molecules dissociated from the aggregates in both micelle (containing 10–30 P1) and unimer forms (Fig. 5 *C*).

Video S2. A representative trajectory of protein-binding-induced disassembly of a P1 nanoparticle The movie was based on simulation 9 from Table S2. P1 polymer is colored gray, and M3 side chains are highlighted in red. bCA-II is shown in green, with Zn ions represented as cyan spheres. DiI molecules are colored with purple.

Importantly, during the dissociation of large aggregates, even as the micelle-micelle interface begins to disassemble and be replaced with micelle-water interfaces, Dil molecules remain well encapsulated and fully shielded from solvent (Fig. 5 *B*), indicating that the hydrophobic cores were not exposed during the initial disassembly process at low protein to polymer ratio (<1:5). This is also consistent with the experimental observations, where a 3:5 molar ratio was required to drive the disassembly and cargo release (15). Additionally, it should be noted that the asphericity index increased significantly just before the assemblies disassembled (Fig. S4). This suggests that the disassembly is likely driven by structural frustration upon specific protein binding.

Indeed, at higher bCA-II to P1 molar ratio (3:2 here), multiple bCA-II molecules can bind to each small micelle, drive further disassembly, and adequately disrupt the hydrophobic core (Fig. 5, *C* and *D*). At this point, the SASA of the DiI head increased up to ∼300–400 A^2^, which suggests that the headgroup is exposed to bulk solvent and the fluorescence would be effectively quenched (Fig. 5 *D* and snapshot III). Due to the highly hydrophobic nature of the DiI tail, the tail of DiI after disassembly remained buried in a small M1 cluster formed out of four to five P1 polymers, consistent with the fact that DiI is insoluble in water. This finding underscores the nuanced nature of the disassembly process, where the initial structural changes lead to the separation of micelles and prepare the aggregates for eventual breakdown, but the full release of the guest molecules occurs only when the integrity of the hydrophobic core is ultimately breached at high protein to polymer ratio. Lastly, it is important to note that the P1 micelles remained stable and did not disassemble even in the presence of high molar ratio of BSA and lysozyme proteins, confirming the specific nature of the binding-induced disassembly response.

### Further validation using the standard Martini 3 model

Since ∼4% of the beads in our model of the P1 polymers and the cargo molecule were assigned nonstandard Martini force field types to capture the chemical specificity of the building blocks (see materials and methods), we conducted additional validation simulations using an alternative model derived strictly according to the standard Martini 3 bead typing guidelines (Fig. S1). Specifically, we assessed potential differences in all three key processes, including self-assembly, cargo encapsulation, and protein-binding-induced disassembly (Figs. S5 and S6).

To examine the self-assembly process, 320 P1 unimers were randomly placed in the simulation box, following the same protocol described earlier for the original model. Starting from a fully dispersed state (cluster number = 0; black trace, Fig. S5 *A*), a single large aggregate rapidly formed within 2 μs (cluster number = 1, Fig. S5 *A*). Consistent with our earlier results (Fig. 2), this aggregate exhibited micelle-like organization with multiple independent hydrophobic cores, as indicated by both the M1-cluster number and the particle distribution profile (Fig. S5 *A*). The process of DiI encapsulation was also examined. During encapsulation, SASA of DiI headgroup dropped from ∼500 A^2^ (in solvent) to less than ∼1 A^2^ within the aggregate (Fig. S5 *B*), consistent with the observations reported in Fig. 4. Notably, the choices of more polar bead types in a couple places (e.g., TN6d for TC1 for the first bead in the linker of Dil) did not appear to have a distinguishable effect on the self-assembly and cargo encapsulation behavior of P1.

Building on this, we further examined the specific protein-binding induced disassembly using the alternative model. Interestingly, a slightly higher protein concentration (25 bCA-II vs. 15 bCA-II in the system containing 80 P1 polymers) and longer simulations (4 μs vs. 1 μs) were required to initiate the disassembly process. This suggests that the P1 aggregates may be slightly more stable due to the use of more hydrophobic beads in a few regions (Fig. 1 vs. Fig. S1). Nonetheless, as summarized in Fig. S6, the essentially same stepwise disassembly mechanism was observed. Also, consistent as our early observation, DiI was released only at the final stage, when the small clusters were disassembled.

Taken together, simulations using the standard Martini 3 model provide a strong validation that the mechanistic insights derived from our model containing a few nonstandard CG bead assignment are reliable. At the same time, we note that these none Martini-standard assignments do not appear to introduce actual improvement despite the rationales. As such, a workflow that adheres closely to the standard Martini 3 guidelines should be more robust and better suited for future studies of similar systems.

## Discussion

We have developed an effective CG modeling framework for direct simulation of the self-assembly and disassembly of nanoparticles formed by P1 amphiphilic polypeptide molecules. The simulations revealed a stepwise assembly of P1 polymers that involves first the formation of small micelle-like structures and then the aggregation of these micelles to form nanoparticles. The final structure of P1 assembly retains the hierarchal organization, and each polymer remains highly dynamic within the assembly. Interestingly, the disassembly process in response to specific protein binding is largely a reverse of self-assembly. The stepwise assembly and disassembly mechanisms highlight the delicate balance between structural stability and responsiveness in these supramolecular assemblies. Furthermore, the release of cargo could potentially be controlled with a high degree of specificity through adjusting the ratio of the specific protein-binding side chains within the polymer structure. By carefully modulating this ratio, it would be possible to regulate the sensitivity of the system to respond to specific protein triggers, thereby controlling the timing and extent of the cargo release. Specifically, in the current system, complete cargo release still requires relatively high concentrations of the target protein. This highlights the need for further optimization to increase sensitivity (e.g., potentially by reducing the intrinsic stability of the assemblies), thereby enabling efficient disassembly and cargo release under biologically relevant conditions.

Another intriguing aspect of the system’s design is the size mismatch between the DiI molecule and the M1 side chain. In the current configuration, the DiI cargo molecule has a bulky hydrophobic tail, which allows both its hydrophilic head and hydrophobic tail to remain fully embedded within the micelles. This unique interaction has the possibility of further fine-tuning the positioning and stability of the cargo by adjusting the head-to-tail volume ratio. By altering this ratio, one could potentially control how deeply the cargo is embedded within the hydrophobic core, thereby influencing the release dynamics (42). Additionally, there is significant potential to further refine the release conditions by introducing protein-specific binding groups directly onto the cargo molecules (43). This strategy could not only enhance the targeting capabilities of the system, allowing for more precise delivery to specific tissues or cells, but also increase the overall safety of the delivery system. For example, by ensuring that the cargo is only released in the presence of its intended target protein, the risk of premature disassembly or release in nontarget areas is minimized. Moreover, this approach could prevent the breakdown of empty vesicles that do not contain cargo, thereby reducing potential side effects. This added layer of specificity could be particularly beneficial in therapeutic applications, where precise targeting and controlled release are critical for efficacy and safety.

It should also be noted that this work builds upon previous efforts on the development of supramolecular assemblies capable of controlled disassembly and cargo release in response to specific biological stimuli, such as protein-binding-triggered disassembly (44,45,46,47,48). The current design, based on structural frustration-driven disassembly, is compatible with other mechanisms of stimulus-induced disassembly. Thus, incorporating secondary or even tertiary triggering mechanisms (e.g., additional responsiveness to enzyme or environmental stimuli such as pH and redox conditions) may significantly enhance the precision, specificity, and stability of conditional cargo release. More broadly, incorporating protein-responsive elements facilitates incorporating responsive characteristics into synthetic P1-like polymers based on a noncovalent binding event, rather than covalent modifications to the polymer. Such a capability opens up new opportunities in designing materials that respond to biological triggers and has implications in a variety of applications including sensing, diagnostics, and target-specific delivery of therapeutics.

The versatility and simplicity of the P1 system, together with its ability to self-assemble and disassemble in response to specific non-covalent binding—and potentially in response to multiple distinct triggers—offers an unprecedented degree of control and may pave the way for advanced materials capable of responding to complex biological environments with high precision. The more detailed understanding of the mechanics of this process acquired from molecular modeling provides valuable insights into the behavior of P1 or similar polymer systems and could inform further experimental or computational studies into the design of more sophisticated and responsive systems that can be finely tuned for specific applications. It is clear that these amphiphilic peptide systems and specific ligands have immense potential for further development and application in various fields of biotechnology and biomedical engineering.

## Conclusion

In this study, we have designed and validated a polymer-based carrier system that conditionally disassembles upon specific protein binding. Through CG simulations, we have elucidated key mechanistic insights into the aggregation, cargo loading, and protein-triggered cargo release processes. The simulations revealed a stepwise dissociation mechanism, highlighting structural frustration and protein-induced destabilization as critical factors driving disassembly. These findings align with experimental observations, suggesting that our computational framework is robust for modeling supramolecular polymer systems. Overall, this work establishes a strong foundation for the rational design of programmable polymers with improved sensitivity, specificity, and stability, facilitating their application in complex biological environments and drug delivery contexts.

## Acknowledgments

The authors thank Stephanie Le for helpful discussions and Jianhan Chen for critical reading of the manuscript. This work was supported by National Institutes of Health grants R35 GM144045 and R35 GM136395.

## Author contributions

Z.J. and S.T. conceived and initiated the studies; Z.J., A.M.C., and S.L. performed molecular modeling, simulations, and analysis; Z.J. and A.M.C. wrote the manuscript; S.L. and S.T. reviewed and edited the manuscript.

## Declaration of interests

The authors declare no competing interests.

## Supporting material

Document S1. Figures S1–S6 and Tables S1 and S2

Document S2. Article plus supporting material
