# Supplementary material for: Stepwise disassembly of supramolecular structures triggered by specific protein binding
Source: Biophys J. 2025 Nov 12;125(2):668–76. doi: 10.1016/j.bpj.2025.11.012 (PMC12688082; doi:10.1016/j.bpj.2025.11.012)
Supplement: Document S1. Figures S1–S6 and Tables S1 and S2 [file mmc1.pdf]

Biophysical Journal, Volume *125*

Supplemental information

Stepwise disassembly of supramolecular structures triggered by specific protein binding

Zhiguang Jia, Allen M. Chen, Shanlong Li, and S. Thayumanavan

Stepwise Disassembly of Supramolecular Structures Triggered by Specific Protein Binding

Zhiguang Jia[1,*], Allen M. Chen[1,2], Shanlong Li[1], and Sankaran Thayumanavan[1,3,*]

[1] Department of Chemistry, University of Massachusetts, Amherst, MA 01003, USA

[2] Northfield Mount Hermon, One Lamplighter Way, Mount Hermon, MA 01354, USA

[3] Department of Biomedical Engineering, University of Massachusetts, Amherst, MA 01003, USA

* Corresponding Author: zhiguangjia@umass.edu (ZJ), thai@umass.edu (ST)

Running title: Protein-binding induced disassembly

Supplementary Tables

Table S1. Sequences of P1 random copolymers simulated

id	sequence
1	GLM1 GLM1 GLM3 GLM2 GLM2 GLM1 GLM2 GLM3 GLM1 GLM2 GLM1
2	GLM1 GLM3 GLM2 GLM3 GLM2 GLM1 GLM1 GLM1 GLM1 GLM2 GLM2
3	GLM1 GLM2 GLM2 GLM3 GLM2 GLM1 GLM1 GLM1 GLM2 GLM1 GLM3
4	GLM1 GLM2 GLM1 GLM2 GLM2 GLM3 GLM1 GLM2 GLM1 GLM1 GLM1

Table S2. Summary of coarse-grained simulations.

**Systems**	**Initial Structure**	**bCA:BSA:lysozyme:DiI** (molecules)	**Boxsize** (Å)	**Length** (μs × runs)
sim 1	10 P1, dispersed	0:0:0:0	160	0.2 × 3
sim 2	20 P1, dispersed	0:0:0:0	160	0.5 × 3
sim 3	40 P1, dispersed	0:0:0:0	200	1.0 × 3
sim 4	80 P1, dispersed	0:0:0:0	280	6.0 × 3
sim 5	160 P1, dispersed	0:0:0:0	320	6.0 × 3
sim 6	320 P1, dispersed	0:0:0:0	360	6.0 × 3
sim 7	80 P1 aggregates, from sim 4	0:0:0:5	280	0.5 × 3
sim 8	10 P1 aggregates, from sim 1	0:0:0:1	160	0.2 × 1
sim 9	80 P1 aggregates with DiI, from sim 7	15:0:0:5	320	1.5 × 3
sim 10	80 P1 aggregates with DiI, from sim 7	0:5:10:5	320	1.5 × 3
sim 11	10 P1 aggregates with DiI, from sim 8	15:0:0:1	250	1.0 × 3
sim 12	10 P1 aggregates with DiI, from sim 8	0:5:10:1	250	1.0 × 3

Supplementary Movies

Movie S1. A representative trajectory of the self-assembly of P1 polymers. The movie was based on simulation 6 from Table S2. P1 polymer is colored grey and M3 sidechains are highlighted in red.

Movie S2. A representative trajectory of protein-binding-induced disassembly of a P1 nanoparticle. The movie was based on simulation 9 from Table S2. P1 polymer is colored grey and M3 sidechains are highlighted in red. bCA-II are shown in green, with Zn ions represented as cyan spheres. DiI molecules are colored with purple.

Supplementary Figures

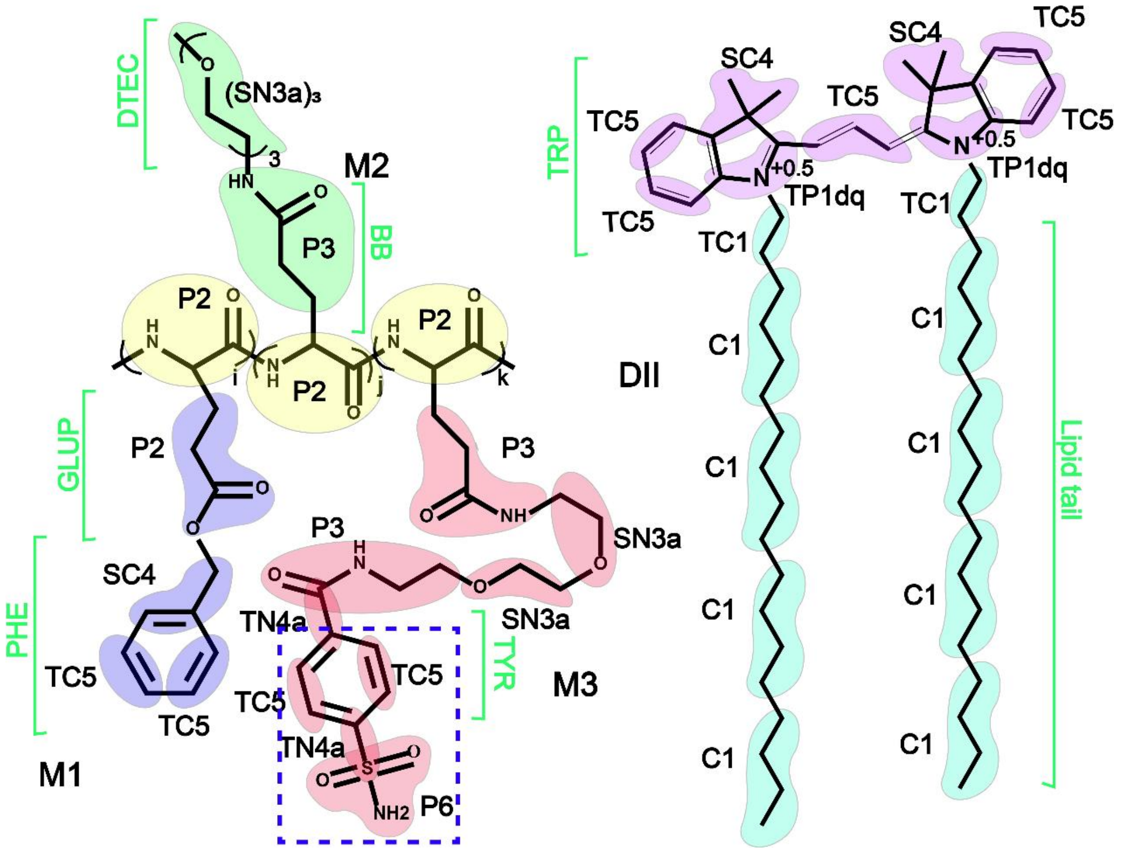

Figure S1. All-atom-to-coarse-grained (AA-CG) mapping of P1 polymers with M1, M2, and M3 monomers (left) and the dye molecule DiI (right) based solely on the standard Martini 3 bead typing guidelines. Clusters of circled atoms are mapped to the same coarse-grain beads and labeled with the corresponding Martini bead types. Overlapping circles indicate atoms contributing to multiple beads. The corresponding building block in Martini 3 is labeled green.

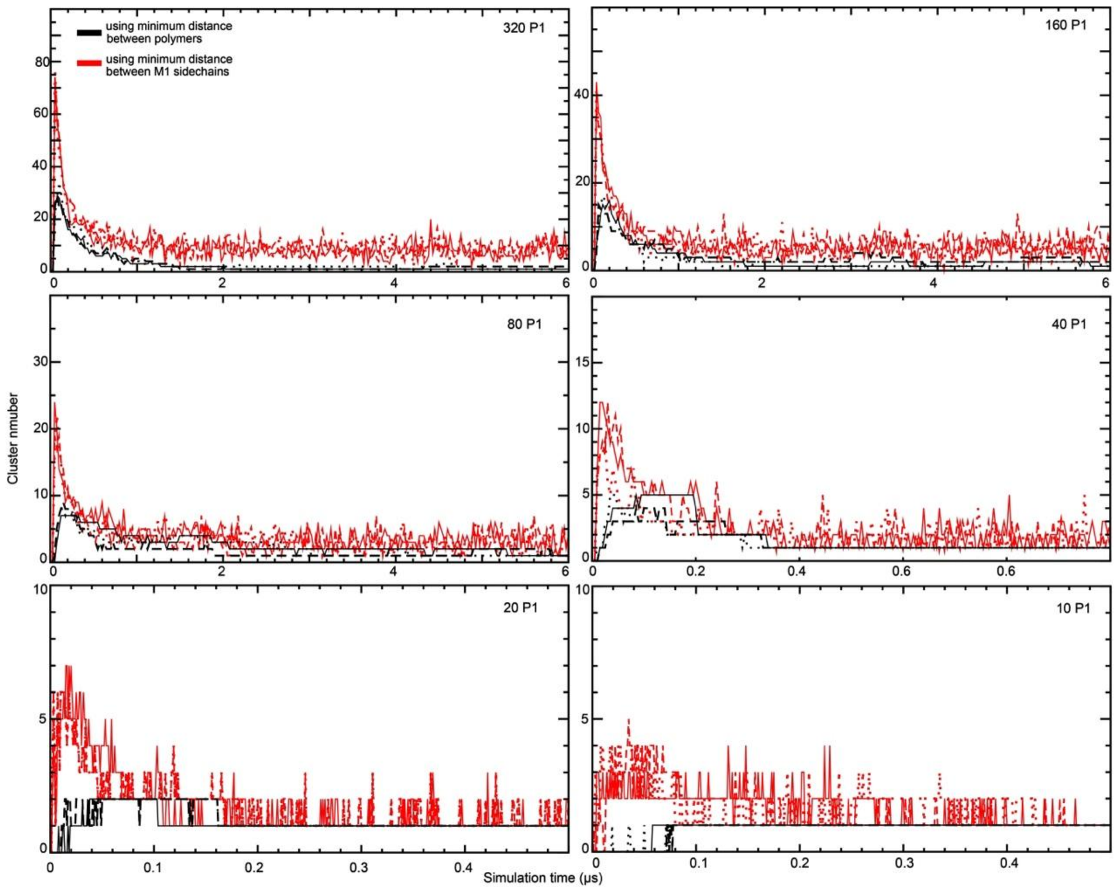

Figure S2. Change in the cluster number as a function of simulation time (Table S2, *sim 1-6*). Average cluster size, calculated using DBSCAN clustering analysis, is plotted as a function of simulation time. Black traces depict calculations using minimum distance between peptides and red traces depict calculations using minimum distance between M1 sidechains.

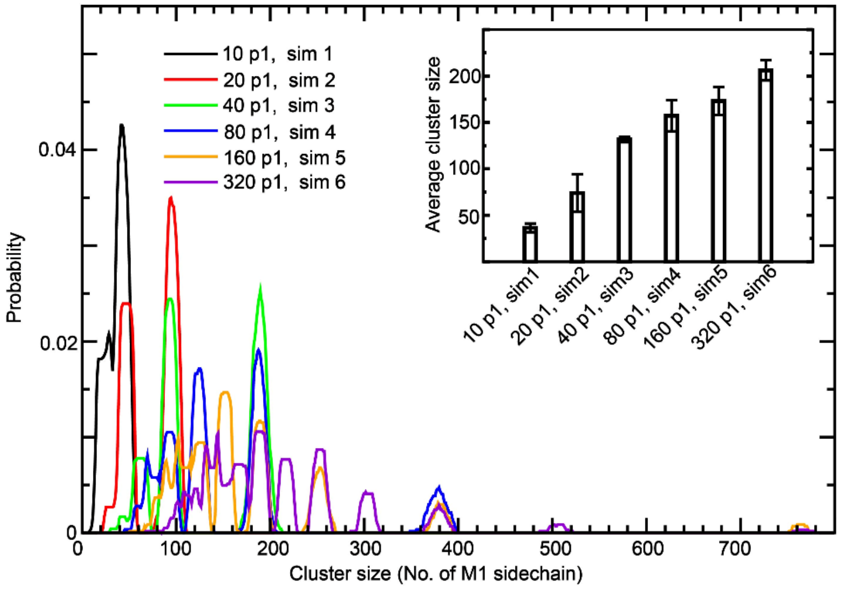

Figure S3. Distribution of cluster sizes for the simulated P1 systems of different sizes, calculated using M1 sidechains. Simulated systems are found on Table S2, *sim 1-6*. The inset figure shows the average cluster size and standard deviation for each system.

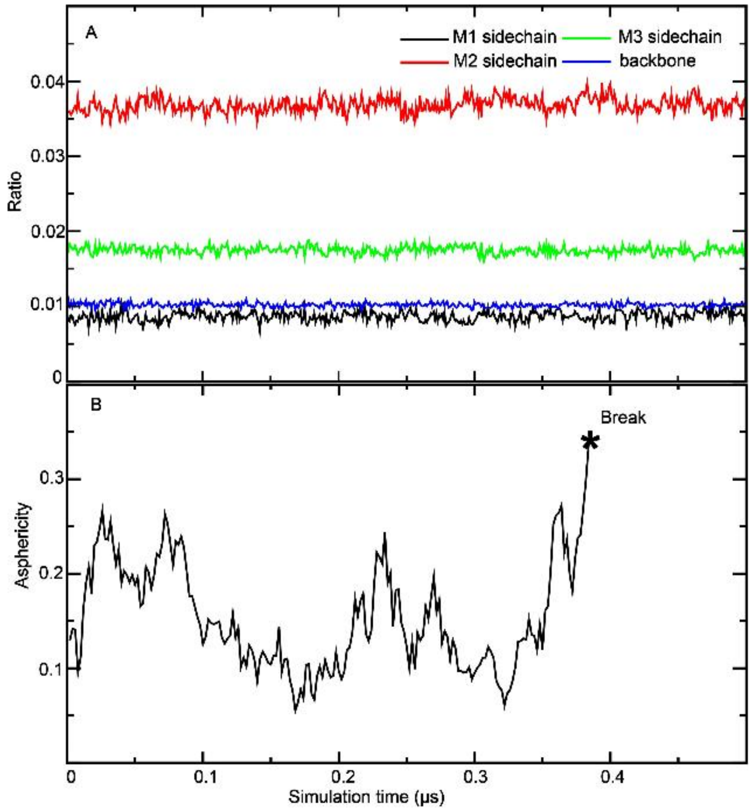

Figure S4. (A) SASA ratio between the four different P1 sidechains and **(B)** asphericity graphed as a function of time during the disassembly simulations for of 80 P1/5DiI aggregates (Table S2, *sim 9*).

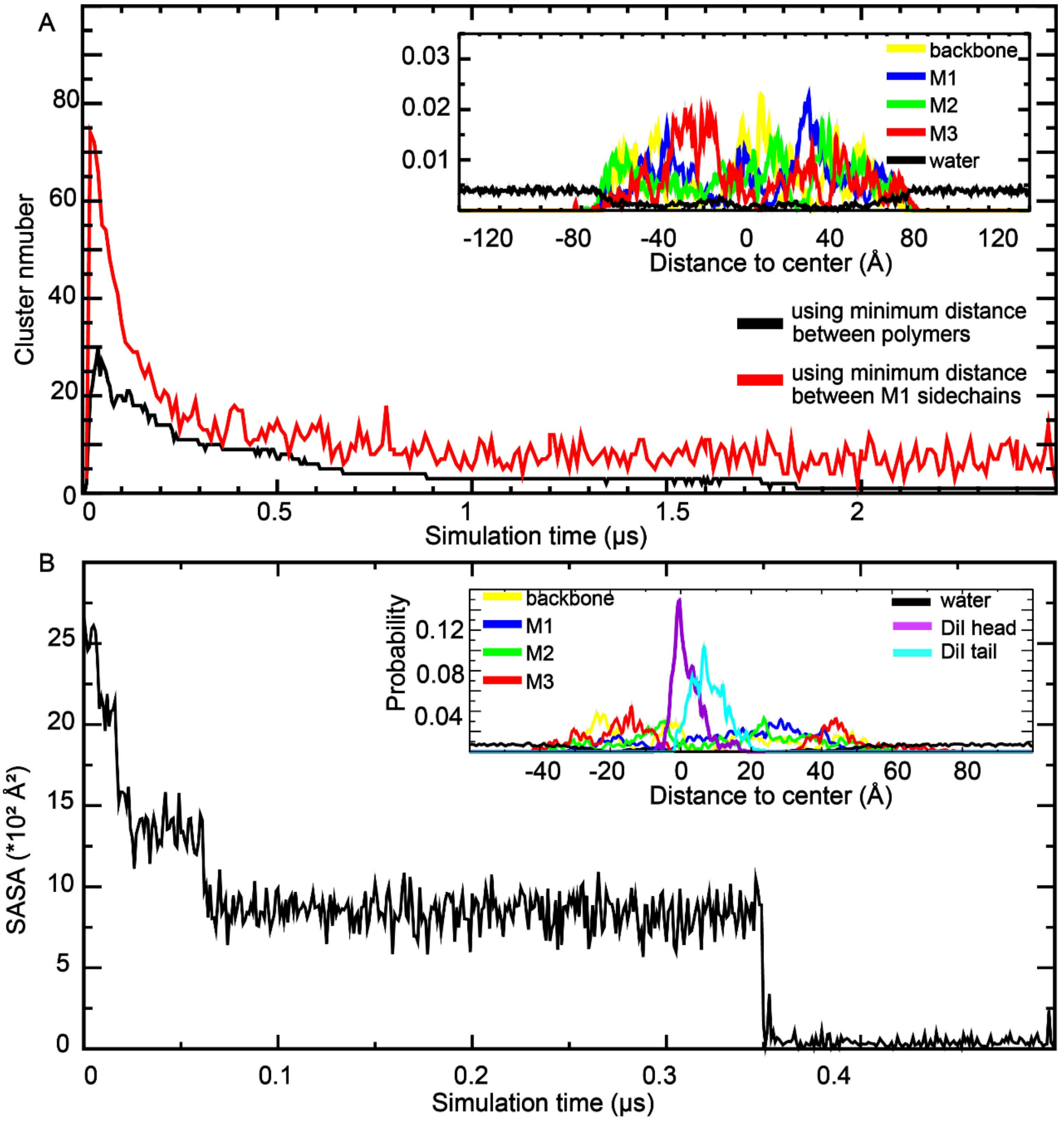

Figure S5: Simulations using the alternative model based solely on the standard Martini 3 bead typing guidelines: (A) Self-assembly of P1 polymers. Change in cluster number as a function of simulation time. The DBSCAN clustering results, using the minimum distance between peptides and M1 sidechains, are plotted as black and red traces, respectively. **(B) Encapsulation of DiI into P1 polymer aggregates.** The solvent-accessible surface area (SASA) of the DiI headgroup is plotted as a function of time. Insert figure shows particle distributions along a cylinder (6 Å diameter) across the center of mass of the 320-mer aggregate or 80-mer aggregate with embedded DiI.

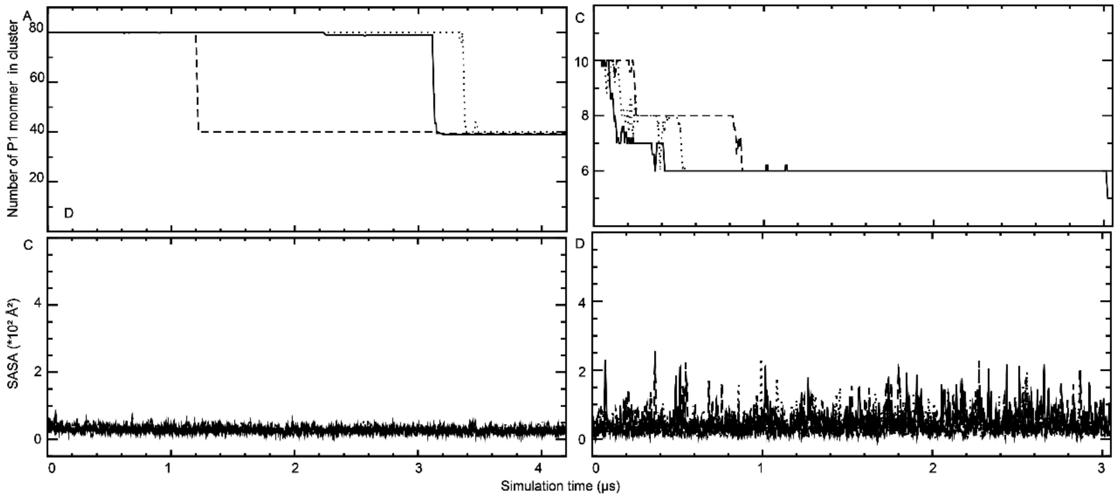

Figure S6: Stepwise disassembly of PI aggregates upon bCA-II binding simulated using the alternative model based solely on the standard Martini 3 bead typing guideline. (A) Average number of P1 polymers in the aggregate and **(B)** SASA of DiI headgroup over time for a system of 80 P1, 25 bCA-II and 5 DiI. **(C)** Number of P1 polymers in a micelle and **(D)** SASA of DiI headgroup over time for a system of 10 P1, 15 bCA-II and 1 DiI. All data are derived from three independent simulations.
